# Acute Suppurative Parotitis Following Acute Ischemic Stroke: A Case Report

**DOI:** 10.7759/cureus.21497

**Published:** 2022-01-22

**Authors:** Junpei Komagamine, Haruka Osawa

**Affiliations:** 1 Internal Medicine, National Hospital Organization Tochigi Medical Center, Utsunomiya, JPN

**Keywords:** ischemic stroke, sialadenitis, geriatrics and internal medicine, infectious disease medicine, suppurative parotitis

## Abstract

Acute suppurative parotitis is an uncommon infection. However, given that acute suppurative parotitis tends to occur in the elderly and considering the aging population, the incidence of acute suppurative parotitis may increase in the future. To prevent acute suppurative parotitis, hydration, good oral hygiene, and avoiding diuretics and anticholinergics are essential. We present the case of a frail elderly patient with acute suppurative parotitis due to dehydration following acute ischemic stroke. Our case highlights the importance of appropriate fluid management in elderly patients.

## Introduction

Acute suppurative parotitis (ASP) is an uncommon infection, and the sudden onset of fever and swelling of the face or neck are typical clinical features. Most patients with ASP are elderly and have severe comorbidities [1]. Common triggers of ASP are dehydration, poor oral hygiene, and the use of drugs such as diuretics and anticholinergics, which reduce salivary fluid. The most common route of infection is retrograde contamination from oral microbial flora [2]. Although many species of bacteria exist in the oral flora, *Staphylococcus aureus* is the most common causative organism, accounting for 30% to 90% of all causative organisms [1-4]. Complications of ASP include abscesses and deep neck infections [5]. Antibiotic therapy can improve this condition in most cases without surgical treatment [2]. However, a substantial proportion of patients with ASP die primarily because of severe comorbidities rather than ASP [1].

This disease has been called “septic parotitis,” “postoperative parotitis,” “surgical parotitis,” and “phlegmonous parotitis” [6]. Although Hippocrates had described epidemic parotitis, whether he recognized ASP is unclear [6]. Therefore, the first description of ASP was likely made by Celsus [6]. In the English literature, ASP was first reported in the Lancet in 1829 [7]. Brodie then distinguished ASP from mumps [8]. In 1868, the first case of postoperative parotitis was reported by Münde [9]. Since then, ASP has been recognized as a severe complication of surgery. In 1881, the American president Garfield died because of ASP after abdominal surgery [5]. In that era, because of inadequate perioperative fluid and electrolyte management and a lack of antibiotics, ASP was a frequent complication of surgery, resulting in death in up to 87% of patients [1]. However, in the 1940s, the advent of antibiotics and improvements in perioperative fluid management has markedly decreased ASP after surgery. In 1955, Robinson stated that ASP was a vanishing disease in surgery [10]. After that, the reemergence of postoperative parotitis due to antibiotic-resistant organisms was reported [11]. However, ASP became an uncommon complication of surgery until the 1970s [5]. Although few studies have investigated the epidemiology of ASP since 1990 [2,5], a recent Danish study also revealed that ASP was uncommon [2]. However, given that one of the most critical risk factors associated with ASP is age [1,12] and considering the aging population, the incidence of ASP may increase in the future. Therefore, knowledge of the optimal diagnosis and management of ASP is crucial. Here, we describe the case of an elderly patient with ASP following acute ischemic stroke.

## Case presentation

**Figure 1:**
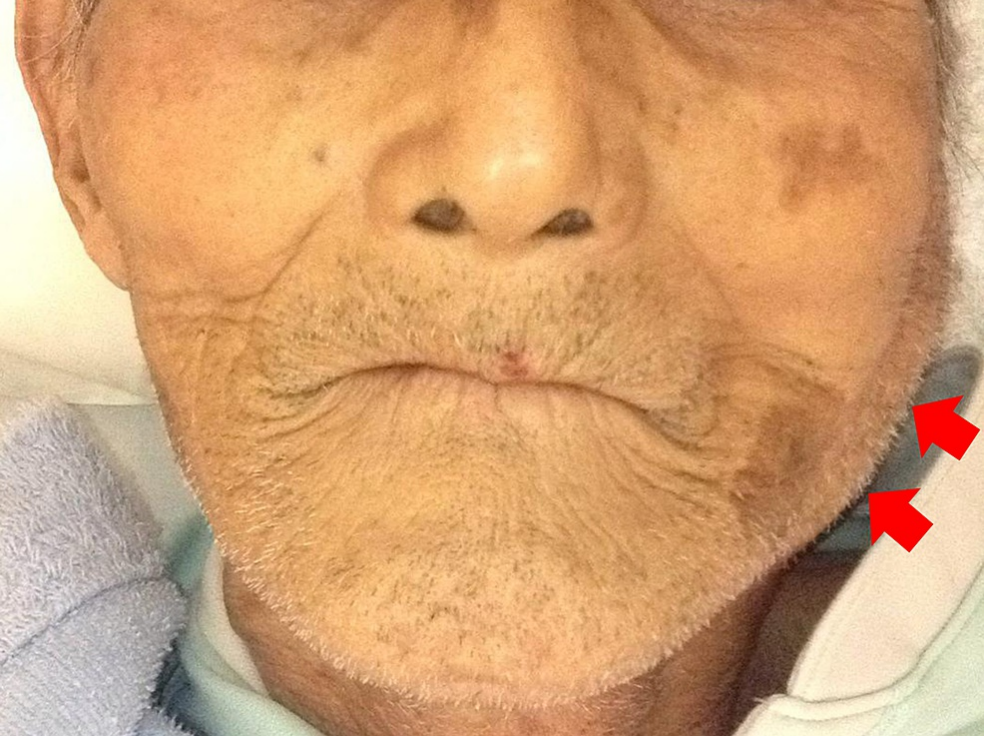
Clinical image showing swelling of the region of the left parotid gland (arrow).

**Figure 2:**
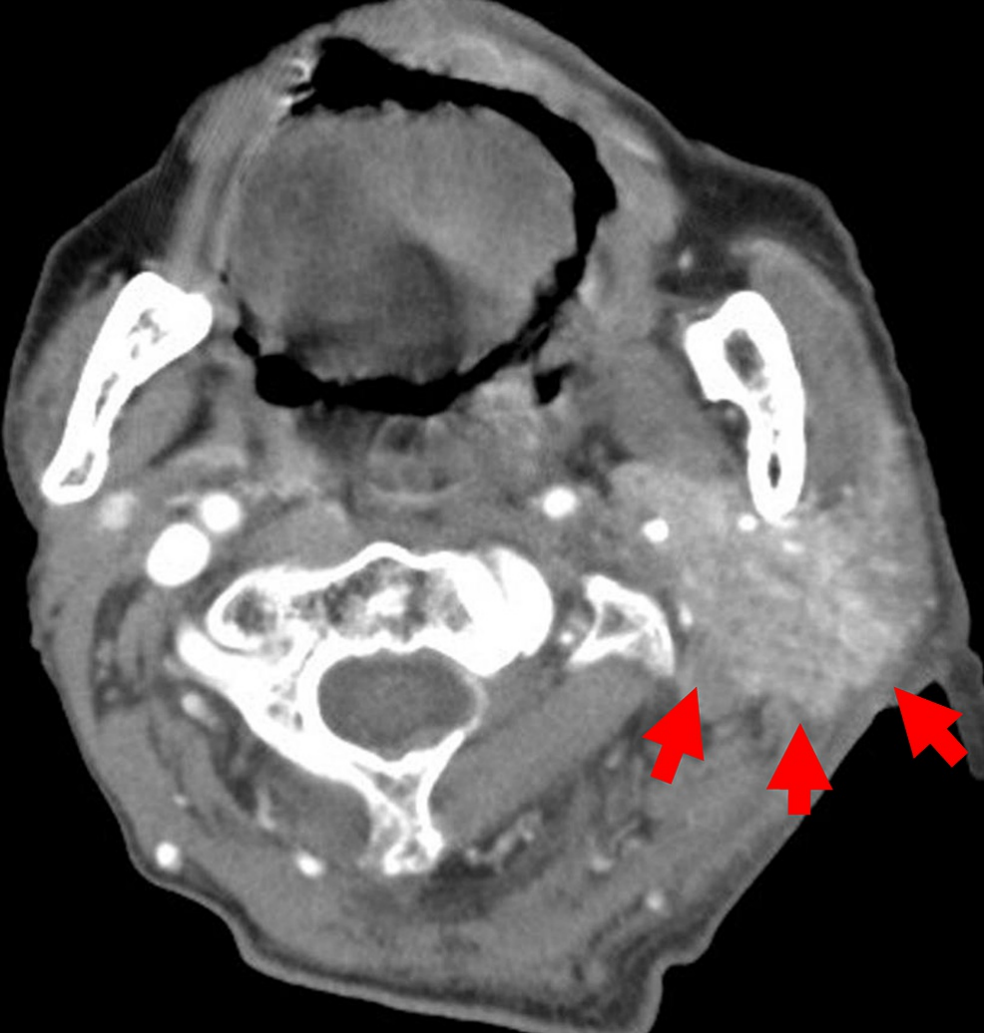
Contrast-enhanced computed tomography (CT) of the neck showing asymmetrical swelling and enhancement of the left parotid gland (arrows).

A 93-year-old man presented to our hospital because of left hemiparesis and dysphagia for three days. He had a history of gastrectomy due to gastric cancer, hip fracture, and chronic obstructive pulmonary disease. He lived partially dependent for walking because of hip fracture. He took nine regular medications, not including diuretics or anticholinergics. Neurological examination revealed left hemiparesis, left sensory impairment, and dysphagia, but the other physical examination findings were not significant. Based on brain magnetic resonance imaging (MRI), acute ischemic stroke involving the right lenticulostriate artery was diagnosed. After admission, aspirin was started, along with rehabilitation. Although his oral intake was poor because of dysphagia after admission, he had received supplementary intravenous fluid at no more than 500 mL per day. On the ninth hospital day, he suddenly developed fever and left neck pain. He was alert and oriented. His temperature was 38.0°C, his blood pressure was 117/90 mmHg, and his pulse was 103 beats per minute. On examination, he had pronounced swelling in the region of the left parotid gland (Figure 1). The tongue and oral mucosa were dry. Pus through the Stensen duct was not apparent, but the assessment was not sufficient because opening his mouth sufficiently was challenging because of neck pain. Laboratory findings revealed leukocytosis, elevated C-reactive protein, and an elevated blood urea nitrogen-to-creatinine ratio (Table 1). Neck contrast-enhanced computed tomography (CT) showed inflammation and asymmetrical enhancement of the left parotid gland (Figure 2). The consulted otolaryngologist diagnosed acute left suppurative parotitis. Initially, ampicillin 8 grams per day was started for treatment. However, on the next day, blood culture revealed methicillin-sensitive *Staphylococcus aureus* (MSSA). Therefore, ampicillin was stopped and then cefazolin 3 grams per day was started. On the 12th hospital day, the fever subsided; on the 13th hospital day, the swelling in the region of the left parotid gland was markedly improved. After the administration of cefazolin for 14 days, ASP was clinically cured. On the 41st hospital day, the patient was transferred to another long-term care hospital because of severe morbidity due to ischemic stroke. Two months later, he died because of aspiration pneumonia at the transfer hospital.

**Table 1:** Summary of the laboratory findings.

Variables	
Hematocrit (%)	45.2
Hemoglobin (g/dL)	15.9
White-cell count (per mm^3^)	26,500
Differential count (%)	
Neutrophils	90
Lymphocytes	4
Monocytes	6
Eosinophils	0
Basophils	0
Platelet count (per mm^3^)	202,000
Sodium (mmol/L)	141
Potassium (mmol/L)	3.5
Chloride (mmol/L)	95
Urea nitrogen (mg/dL)	19.3
Creatinine (mg/dL)	0.6
Creatine kinase (U/L)	253
Glucose (mg/dL)	131
Alanine aminotransferase (U/L)	18
Alanine aminotransferase (U/L)	12
C-reactive protein (mg/dL)	19.1

## Discussion

This case was of a frail elderly patient with ASP due to dehydration following acute ischemic stroke. Although the patient recovered from ASP, he died because of aspiration pneumonia due to dysphagia within three months after ASP onset. This case provides several important lessons. First, appropriate fluid management is critical to prevent ASP because one of the most common triggers of ASP is dehydration [1,5,12]. In our case, despite poor oral intake due to dysphagia, the dose of fluid infusion per day was low. This suboptimal fluid management might result in dehydration. At the disease onset, the patient’s oral mucosa was dry, and the blood urea nitrogen-to-creatinine ratio was elevated beyond 30 in laboratory tests. Given that an elevated blood urea nitrogen-to-creatinine ratio was associated with a longer duration of hospital stay [13], maintaining a blood urea nitrogen-to-creatinine ratio of less than 20 in addition to monitoring for physical signs of dehydration is important to prevent or treat ASP.

Second, given that most causative organisms of ASP are *S. aureus*, *Streptococcus spp*., and anaerobes [1,3,5], immediate empirical antibiotic therapy targeting these organisms is important. A previous study reported that antibiotic use, which is effective for causative organisms, is associated with a lower risk of death [1]. Given that a substantial proportion of frail elderly patients with ASP died within 72 hours of the onset [14,15], appropriate initial empirical antibiotic therapy is critical. In our case, ampicillin was initially prescribed for presumed streptococcus or anaerobe infections of the left parotid gland. However, blood culture isolated MSSA, and then, this resulted in changing the antibacterial drug from ampicillin to cefazolin. Although this delay of appropriate antibacterial therapy did not lead to an immediate death, ampicillin-sulbactam at 12 grams per day should be selected as the initial empirical antibiotic therapy.

Finally, as some authors noted, ASP may indicate a poor prognosis for elderly patients [14,15]. Although a few studies have investigated the clinical features of ASP in frail elderly patients [14], several small case series have shown high mortality rates among these patients [14,15]. In elderly patients with ASP, most causes of death are background diseases rather than ASP itself, while otherwise healthy young patients with ASP sometimes recover spontaneously without antibiotic use or surgical therapy [16]. In our case, the patient died because of aspiration pneumonia rather than ASP within three months after its onset.

A recent retrospective study reported that an elevated blood urea nitrogen-to-creatinine ratio and elevated level of inflammatory markers, such as white cell count and C-reactive protein, are associated with a longer duration of hospitalization among patients with ASP [13]. In fact, in our case with elevated levels of all these markers, the duration of hospital stay was long.

Although ASP is an uncommon infection, its incidence may increase in the future because of the aging population. Given that elderly patients easily become dehydrated and are often prescribed anticholinergics and diuretics [12], appropriate fluid and drug management are critical to prevent ASP. Awareness of preventive and management strategies for ASP may help mitigate the reemergence of ASP in elderly patients.

## Conclusions

Although ASP has become a rare complication in operative patients, its incidence may increase in frail elderly patients. The most common trigger is dehydration, as in our case. Therefore, avoiding dehydration is crucial to prevent ASP. Additionally, early appropriate antibiotic therapy is critical to improve the prognosis of patients with ASP. Given that the most common causative organisms are *S. aureus*, *Streptococcus spp*., and anaerobes, antibiotics that are effective against them should be selected initially for ASP. Given that ASP is an uncommon disease, physicians' awareness of preventive and management strategies for ASP is crucial.
